# A customizable software for fast reduction and analysis of large X-ray scattering data sets: applications of the new *DPDAK* package to small-angle X-ray scattering and grazing-incidence small-angle X-ray scattering

**DOI:** 10.1107/S1600576714019773

**Published:** 2014-09-30

**Authors:** Gunthard Benecke, Wolfgang Wagermaier, Chenghao Li, Matthias Schwartzkopf, Gero Flucke, Rebecca Hoerth, Ivo Zizak, Manfred Burghammer, Ezzeldin Metwalli, Peter Müller-Buschbaum, Martin Trebbin, Stephan Förster, Oskar Paris, Stephan V. Roth, Peter Fratzl

**Affiliations:** a PETRA III, Deutsches Elektronen-Synchrotron (DESY), Notkestrasse 85, Hamburg 22607, Germany; b Department of Biomaterials, Max Planck Institute of Colloids and Interfaces, Am Mühlenberg 1, Potsdam, Brandenbug 14476, Germany; c Berlin-Brandenburg School for Regenerative Therapies (BSRT), Charité – Universitätsmedizin Berlin, Augustenburger Platz 1, Berlin 13353, Germany; d Institut für Nanometeroptik und Technologie, BESSY II, Helmholtz-Zentrum Berlin für Materialien und Energie (HZB), Albert-Einstein-Strasse 15, Berlin 12489, Germany; e ID13, European Synchrotron Radiation Facility (ESRF), 6 Rue Jules Horowitz, Grenoble 38000, France; f Department of Analytical Chemistry, Ghent University, Krijgslaan 281, S12, B-9000 Ghent, Belgium; g Lehrstuhl für Funktionelle Materialien Pysik Department E13, Technische Universität München, James-Franck-Strasse 1, Garching 85747, Germany; h University of Bayreuth, Physical Chemistry 1, 101251, Bayreuth 95440, Germany; i Institut für Physik, Montanuniversität Leoben, Franz-Josef Strasse 18, Leoben 8700, Austria

**Keywords:** small-angle X-ray scattering, grazing-incidence small-angle X-ray scattering, data reduction, data analysis, computer programs

## Abstract

X-ray scattering experiments at synchrotron sources are characterized by large and constantly increasing amounts of data. The great number of files generated during a synchrotron experiment is often a limiting factor in the analysis of the data, since appropriate software is rarely available to perform fast and tailored data processing. Furthermore, it is often necessary to perform online data reduction and analysis during the experiment in order to interactively optimize experimental design. This article presents an open-source software package developed to process large amounts of data from synchrotron scattering experiments. These data reduction processes involve calibration and correction of raw data, one- or two-dimensional integration, as well as fitting and further analysis of the data, including the extraction of certain parameters. The software, *DPDAK* (directly programmable data analysis kit), is based on a plug-in structure and allows individual extension in accordance with the requirements of the user. The article demonstrates the use of *DPDAK* for on- and offline analysis of scanning small-angle X-ray scattering (SAXS) data on biological samples and microfluidic systems, as well as for a comprehensive analysis of grazing-incidence SAXS data. In addition to a comparison with existing software packages, the structure of *DPDAK* and the possibilities and limitations are discussed.

## Abstract 2

*DPDAK* is a software for simple and fast on- and offline reduction and analysis of X-ray scattering data. It is an open-source software with a plug-in structure allowing tailored extensions.

## 1. Introduction   

High-brilliance synchrotron radiation sources allow today’s use of X-ray scattering methods with a spatial resolution in the range of micro- and even nanometres (Riekel, 2000 ▶; Paris, 2008 ▶; Narayanan, 2009 ▶). High-resolution scanning small- and wide-angle X-ray scattering (SAXS/WAXS) techniques are frequently used for scanning across thin sample sections to map local structural features in heterogeneous systems at length scales that are not accessible by measurements of single frames (Paris, 2008 ▶; Gourrier *et al.*, 2007 ▶). Another powerful scattering technique is grazing-incidence small-angle X-ray scattering (GISAXS), providing information with high statistical significance on the nanostructure of very small sample volumes in thin-film geometry (Müller-Buschbaum, 2003 ▶). The high photon flux at synchrotron sources allows the fast performance of scattering experiments as well as the carrying out of experiments that involve fast timescales. Current detector generations with frame rates of up to 1000 Hz and readout times below 1 ms together with high position resolution lead to very high data rates and subsequently often to a high number of recorded frames (Ponchut *et al.*, 2011 ▶; Trueb *et al.*, 2012 ▶). Such large amounts of data require dedicated software tools for viewing, reduction and analysis.

Quantitative analysis of small-angle scattering data often requires structural models or the incorporation of additional information. A large amount of effort has been put into establishing packages for quantitative analysis, especially of scattering data from emulsions or solutions to extract low-resolution protein structures (Svergun *et al.*, 2013 ▶) or the size and shape of colloids and particles (Glatter & Kratky, 1982 ▶). The input for such analysis programs is clean background-corrected SAXS or SANS (small-angle neutron scattering) data. Data reduction and simple analyses, such as the extraction of invariants (Porod, 1951 ▶) of the SAXS or SANS curve, are needed to pre-screen the data and have to be carried out before in-depth analyses, often with large amounts of data and in an online fashion directly during the data collection to allow for potential modifications of the experimental strategy.

This leads to the following four essential key features for a fast on- or offline data reduction and analysis software: (i) an easy adaptation to specific needs; (ii) an online mode enabling a pre-analysis of data in parallel to measurements, with automatic handling of new data as well as the ability to deal with high data rates; (iii) an open-source philosophy, which is essential to verify and extend the functionality and to allow the software to be maintained by a larger group; and (iv) support of Windows and Linux operating systems. The latter is necessary because most beamlines use Linux for their experiment control, but many users work on Windows PCs. Nowadays there are several tools available; some are listed in Table 1 ▶ (for more see *e.g.* http://smallangle.org/content/software). Most of the reviewed tools are open source and run on both Windows and Linux computers, but either do not have an online mode or have no plug-in or macro interface.

In this article, we present a new directly programmable data analysis kit (*DPDAK*), which includes all required features. *DPDAK* is now in use at the micro- and nanofocus X-ray scattering beamline at PETRA III (MiNaXS P03) and the μSpot SAXS/WAXS beamline at BESSY II (HZB, Berlin, Germany). We provide detailed information about the main features of *DPDAK*. *DPDAK*’s framework structure is described and the plug-in interface is elucidated so that the software can be extended with new features. Finally, we demonstrate how *DPDAK* can be used for online analysis of scanning SAXS/WAXS experiments and *in situ* GISAXS sputter deposition experiments.

## 2. Specifications and features   

**Figure 1:**
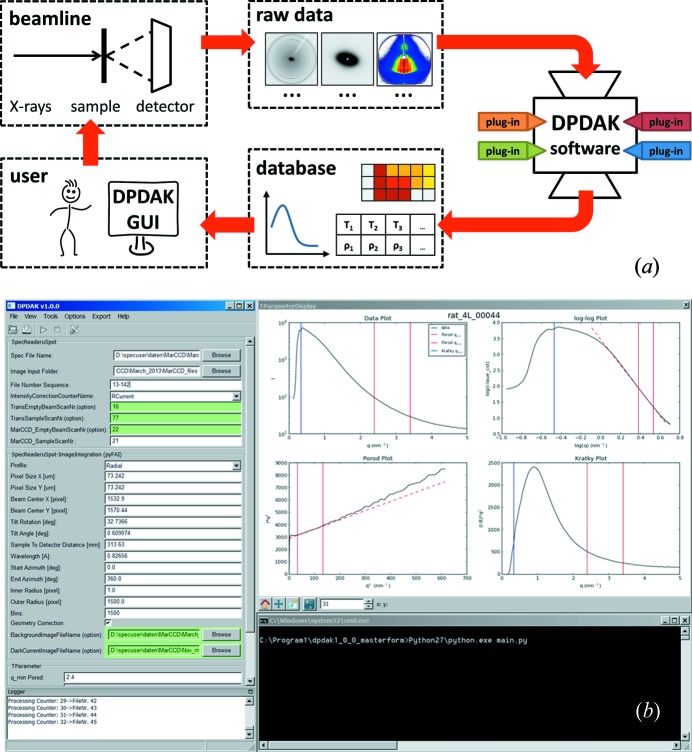
(*a*) Work- and dataflow during a synchrotron experiment with online data analysis by *DPDAK*. Devices at the beamline produce a constant raw data flow. The raw data (detector images, motor positions) are read instantly by the *DPDAK* framework. Depending on the used plug-ins, a resulting data set of reduced data (curves, maps, scalar values *etc*.) is produced and stored in a database. The results can be accessed by the user *via* the GUI. (*b*) Screenshot of *DPDAK* during online analysis of SAXS on bone at the BESSY μSpot beamline (data presented in Fig. 2 ▶). The left side shows the user interface with file path, sequence and several evaluation parameters. The right side shows four plots representing details of the evaluation of the SAXS data: upper left is a radial integration, upper right the same data in a log–log plot, lower left shows a Porod plot and lower right a Kratky plot.

*DPDAK* is based on a plug-in framework and is currently used as a tool for analysis of WAXS, SAXS and GISAXS data. The software package is written in Python and the motivation for this is based on four major facts: (i) Python is open source and is (ii) well established in the experimental physics scientific community, which results in (iii) a huge variety of packages of algorithms and mathematical tools. Compared to other programming languages, Python has (iv) a simpler syntax, which makes it easier to use for non-computer scientists. The user interface allows for plotting of one-dimensional curves, two-dimensional color maps and scattering images. All plots can be navigated by zooming and panning and allow the range and scaling on the axis to be changed (see screenshot in Fig. 1 ▶ *b*). Current export plug-ins allow for saving data in text files in spreadsheet format. Image sequences can be converted to other file formats or saved as video files. Most of the plug-ins read and operate on images, curves and numerical data. They are processed sequentially to enable online analysis during current measurements. For reading diffraction images *DPDAK* uses the *FabIO* Python library (Knudsen *et al.*, 2013 ▶). It supports a wide range of file formats like tif, mar, edf and cbf and can be extended with new formats in a simple way. *DPDAK* uses the Python packages *NumPy* and *SciPy* (http://www.scipy.org/) for numerical operations and fitting. The graphical user interface (GUI) is implemented using the package *wxPython* (http://www.wxpython.org/). *Matplotlib* is used to display any kind of graphs from one-dimensional to two-dimensional plots. The *DPDAK* Windows version includes a Python installation with all these packages, while the Linux version requires a manual installation. Scattering images can be corrected for background and intensity and binned before integration. Integration is implemented as simple box integration for peak and median intensity profiles, various GISAXS line cuts, and SAXS integration for azimuthal and radial profiles. A peak fitting tool for one-dimensional data can be used for data with an arbitrary combination of Gaussian, Lorentzian and pseudo-Voigt profiles and background functions.

**Figure 2:**
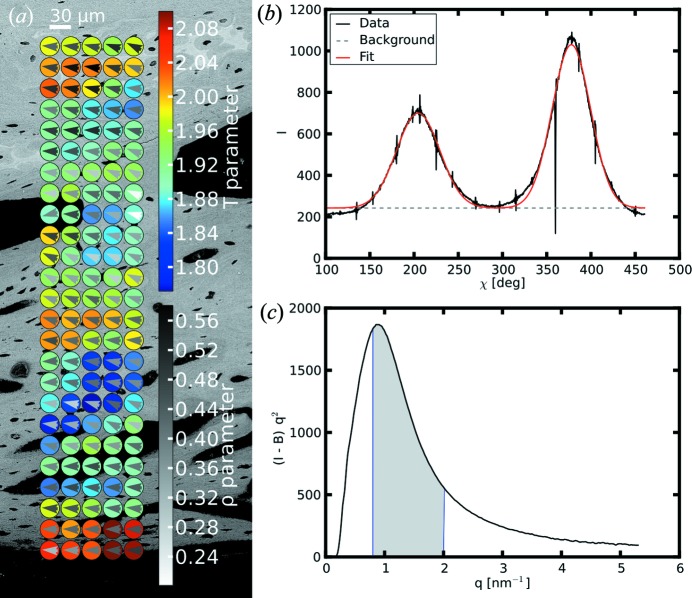
Results of processed SAXS data collected at the μSpot beamline, BESSY II, HZB. (*a*) Callus of a rat bone: SEM image (BSE) with *T* (color-coded circles) and ρ parameters (gray level of the triangles within the *T* circles), and predominant particle orientation (direction of gray triangles). (*b*) Azimuthal intensity distribution for determination of the ρ parameter. (*c*) Kratky plot for determination of the *T* parameter.

*DPDAK* features include integration and calibration of SAXS and WAXS scattering patterns, which is implemented by using either *Fit2D* (Hammersley *et al.*, 1996 ▶) or the *pyFAI* library (Kieffer & Karkoulis, 2013 ▶). The user can choose between those two methods by selecting the corresponding plug-in – although we recommend the *pyFAI* method since it is more comprehensible and faster than *Fit2D*. By combining the *pyFAI* library from ESRF (European Synchrotron Radiation Facility in Grenoble, France) with the *DPDAK* GUI, we suggest that this may replace *Fit2D*, which is no longer being developed. For GISAXS setups, *DPDAK* allows users to create horizontal line cuts (called out-of-plane cuts) along 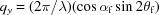 and vertical line cuts (called detector cuts) along 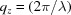
 × 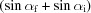, as well as along arbitrary directions in *q_y_*, *q_z_*. Cuts can be integrated over a region of interest (ROI) for better statistics (Schwartzkopf *et al.*, 2013 ▶). *DPDAK* includes a SAXS-based *T* and ρ parameter calculation, mainly used for structural bone analysis. The ρ parameter is a measure of the degree of alignment of the mineral platelets within the irradiated bone volume (Pabisch *et al.*, 2013 ▶; Rinnerthaler *et al.*, 1999 ▶). The predominant orientation of the mineral particles is calculated from the anisotropy of the SAXS patterns by computing azimuthal *q* averages of the intensity, resulting typically in a profile with two peaks that are separated by 180° for anisotropic particle orientations (see Fig. 2 ▶ *b*). *DPDAK* fits the peaks and calculates the ratio between the area under the peaks and the background. The *T* parameter is a measure of the thickness of mineral particles (Fratzl, Schreiber & Boyde, 1996 ▶; Fratzl *et al.*, 1991 ▶) and can be calculated by using Porod’s law (Porod, 1951 ▶).

## 3. Workflow and framework structure   

*DPDAK* is designed as a flexible online data processing toolkit. An illustration of *DPDAK*s work- and dataflow is given in Fig. 1 ▶(*a*). During an experiment at a synchrotron the user controls the beamline *via* the on-site infrastructure, and raw data in the form of scattering patterns are produced and stored. A *DPDAK* installation runs in parallel to read the raw data directly from a file system and to perform user-defined data reduction processes. The results are stored in the *DPDAK* database and can be displayed in various forms (images, plots *etc*.) to the user. Fig. 1 ▶(*b*) shows a screenshot of *DPDAK* during online analysis of SAXS on bone (the data after evaluation are presented in Fig. 2 ▶). The left side shows the user interface with the file path and several evaluation parameters. The right side shows four plots representing details of the evaluation of the SAXS data (for details see the applications example in Fig. 2 ▶). Similar or more complex and accurate analysis of raw data can be performed after the experiment with the user’s own ‘offline’ *DPDAK* installation.

*DPDAK* is implemented in a framework structure based on plug-ins (small program units). The basic concept is a model–view–controller (MVC) architecture pattern (Krasner & Pope, 1988 ▶). This approach aims to separate the code for the user interface from the application data and the code for user interaction and processing. As a result of this separation the code becomes more structured into specialized individual components, and the application becomes independent of its GUI. The controller implements the data processing, where plug-ins can be connected in a tree-like structure. The data are sent from plug-in to plug-in and each plug-in adds new data to the output. For each incoming data segment (*e.g.* detector image) the plug-in structure is processed.

The *DPDAK* framework implements an easy to use storage for all processed data, called the database. For fast access, all processed data are held in memory, except for image files which are linked to their location on a hard drive. The database is implemented as a table-like structure. Each column corresponds to a plug-in output and has therefore a defined type (*e.g.* a scalar value or a curve). Each row corresponds to a data set, the complete output of one or more plug-ins at a specific time.

## 4. Programming user-defined plug-ins   

Since Python is an interpreted programming language, developing a plug-in system is possible without the implementation of a scripting language or the need to compile plug-ins. By using the built-in function _import_(name), a text file can be imported as a Python module just by its file name. Therefore, all plug-ins are stored in a plug-in subfolder inside the main program folder and are imported at program start. A new plug-in can be added by creating a text file with the .py extension inside the plug-in folder.

The *DPDAK* framework distinguishes three types of plug-ins: base, display and export plug-ins. A base plug-in is used to read data (detector images, text files *etc*.) and process data. The processing of data can vary from simple arithmetic operations up to fitting a model function. Display plug-ins add new elements to the GUI. They have access to the database to display data, *e.g.* a plot. An export plug-in can be used to export data from the database to any kind of format.

**Figure 3:**
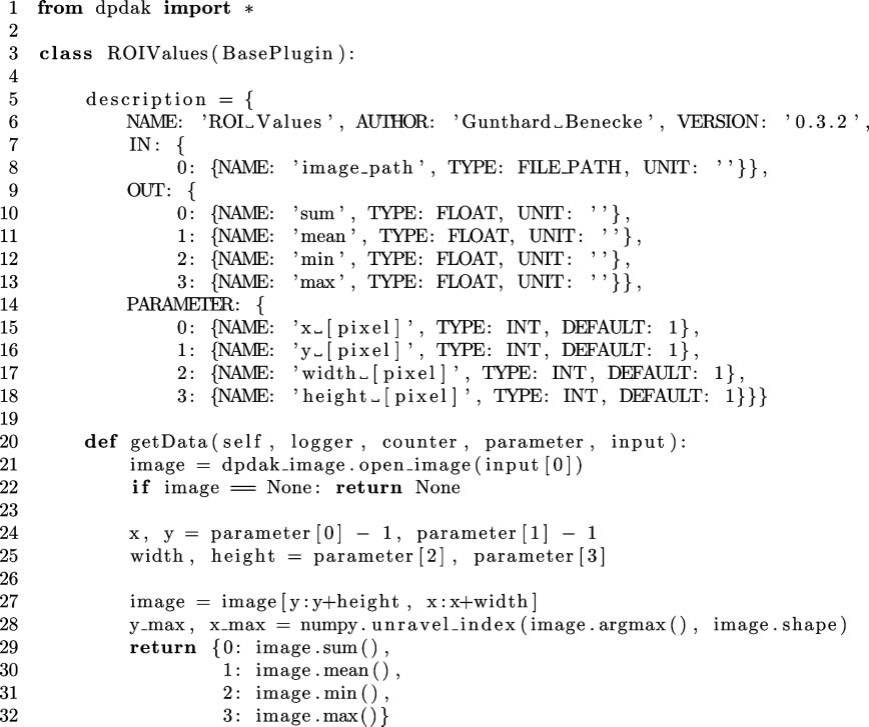
Example code for a plug-in calculating the sum, the average, the minimum and the maximum intensity of a rectangular region inside a detector image.

In order that the framework can work with a plug-in, it must make use of a defined interface. There exists one Python class for each plug-in type. Those classes contain some methods that must be implemented to make the plug-in work. As most of the users will mainly add base plug-ins, in Fig. 3 ▶ we show as an example the code for a plug-in calculating the minimum, maximum, average and sum intensity of an ROI in a detector file. In the description class variable (lines 5–18), the required parameters and the in- and output of the plug-in are defined. Parameters are inputs for the users to control the plug-in. Each defined parameter is automatically generated and shown in the GUI. In the example (Fig. 3 ▶), parameters for the position, width and height of the ROI are defined. The input and output information tells the framework which data are needed by the plug-in and which new data will be generated and have to be stored in the database. In this case a detector image is requested as input data and four scalar values are returned as output. Lines 20–32 show the implementation of the getData method, which is called for each data set in the database. The parameters and input are used to calculate the output values. The method needs to return the data as defined.

## 5. Results and discussion of application examples   

**Figure 4:**
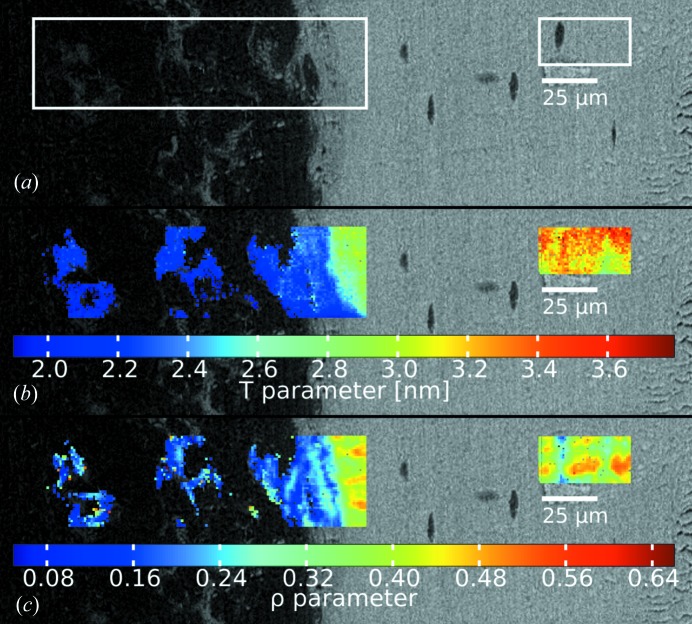
Results of processed SAXS data collected at ID 13, ESRF, with a micrometre sized beam. (*a*) Mouse bone: femur with an osteotomy, imaged with BSE. (*b*) *T* parameter map. (*c*) ρ parameter map.

In order to give an overview of *DPDAK*’s capabilities, we present five examples of applications. First we show two examples where the nanostructure of biological bone samples is determined by scanning SAXS and WAXS experiments at the μSpot beamline at BESSY II (HZB, Berlin, Germany) and at the microfocus beamline ID 13 at ESRF (Grenoble, France). This is followed by two experiments performed at the MiNaXS/P03 beamline at PETRA III (DESY, Hamburg, Germany) which highlight the online analysis of GISAXS experiments to track sputtering of gold on silicon and polymer substrates. The fifth experiment (done at the MiNaXS/P03 beamline at PETRA III) shows a mapping of SAXS data visualizing the flow of polymeric wormlike micelles in microchannels. These examples reflect the following advantages of *DPDAK*: (i) precise evaluation of data sets with several tens of thousands of data points is only achievable by using tailored plug-ins extracting the expedient parameters (see application examples in Figs. 4 ▶, 5 ▶ and 6 ▶), (ii) online analysis of SAXS and WAXS data during scanning enables a fine tuning of measurement parameters as well as an optimized selection of ROIs (see application examples in Figs. 2 ▶, 7 ▶ and 8 ▶), (iii) online analysis of GISAXS data gives immediate feedback if sputtering experiments are successful (see application example in Fig. 7 ▶), and (iv) fast online generation of two-dimensional maps of reduced diffraction data from microflow experiments allows a control of experimental parameters (see application example in Fig. 8 ▶).

Bone is an excellent example of a biphasic biological material, exhibiting an electron density difference due to mineral particles embedded in an organic matrix. SAXS can give information on the mean thickness (*T* parameter) and the degree of orientation (ρ parameter) of the mineral particles, as well as their predominant orientation (Fratzl, Schreiber & Klaushofer, 1996 ▶; Rinnerthaler *et al.*, 1999 ▶), while WAXS gives information on the crystal lattice of the hydroxyapatite platelets (crystal length and orientation of the crystalline *c* axis) (Pabisch *et al.*, 2013 ▶). After fracture, bone is able to heal, and during healing the bone material undergoes various changes which can be monitored by visualizing the above-mentioned parameters. Fig. 2 ▶(*a*) shows a small region of a callus of an osteotomized rat femur (cut through the bone to induce a healing response) during healing four weeks post-osteotomy, imaged with scanning electron microscopy in backscattered electron mode (BSE). A thin slice (around 70 µm) of the sample was investigated at the μSpot beamline at BESSY II using scanning SAXS with a 30 µm beam. The evaluation of the *T* and ρ parameters was performed in online mode with *DPDAK* during the measurement, which enabled a fine tuning of measurement parameters (scanning step size, *q* range, exposure time *etc*.) and a tailored choice of the most interesting measurement regions. Fig. 1 ▶(*b*) shows a screenshot of *DPDAK* during the online evaluation. These data were used to display Figs. 2 ▶(*b*) and 2 ▶(*c*), which show the azimuthal intensity profile and the Kratky plot that are calculated by *DPDAK*. These can be displayed for each measurement point separately to control and fine tune the evaluation procedure. The colored circles in Fig. 2 ▶(*a*) show the measurement points, where the color corresponds to the average particle thickness *T* and the gray triangles represent their preferred orientation, while the gray scale of the triangles refers to the ρ parameter (degree of alignment).

Another bone sample was measured at beamline ID13 at ESRF with a 1 µm beam in scanning mode. Fig. 4 ▶(*a*) shows a BSE image of a mouse bone with an osteotomy, and the white rectangles define the ROIs, where scanning SAXS was performed. Fig. 4 ▶(*b*) displays these ROIs of the BSE image overlaid by a map of *T* parameters, where every pixel corresponds to a single SAXS measurement point. The image consists of 9012 frames, and the *T* and ρ parameter evaluation was performed offline using the corresponding plug-ins in *DPDAK*.

Grazing-incidence small-angle X-ray scattering is one of the most powerful methods to investigate *in situ* the growth of metallic layers during vacuum deposition. With third-generation synchrotron sources, high-rate sputter deposition (0.21 nm s^−1^) and fast data acquisition have become a standard tool for surface science. We present as an example the growth of gold clusters during sputter deposition on a silicon substrate (Schwartzkopf *et al.*, 2013 ▶), which exemplarily shows the challenges for the software and the rapid analysis. The experiment was performed at beamline MiNaXS/P03 of DESY (Buffet *et al.*, 2012 ▶). During the experiment, 10 000 frames were acquired by a PILATUS 300K (DECTRIS Ltd) detector in 150 s (66.67 frames per second) using a 38 × 19 µm microfocused beam with a wavelength of λ = 96.0 ± 0.2 pm at an incident angle of α_i_ = 0.5° and a sample-to-detector distance of *D*_SD_ = 2750 ± 2 mm.

**Figure 5:**
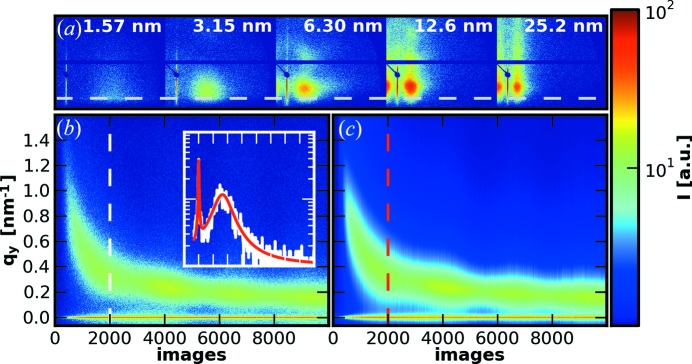
(*a*) Five representative GISAXS patterns from thin (left) to thick (right) Au layers. The gray dashed line indicates the *q_y_* position of the Yoneda peak of silicon. (*b*) Map of extracted horizontal line cuts at the Yoneda peak position of silicon as a function of the frame number. The inset shows an individual fit of frame 2000, indicated by the white (data) and red (fit) dashed lines. (*c*) Map of the corresponding fitted horizontal line cuts. The logarithmic intensity scale bar applies to all figures.

In Fig. 5 ▶(*a*) we present a selection of GISAXS patterns at the deposited film thicknesses of 1.57, 3.15, 6.3, 12.6 and 25.2 nm, corresponding to frame numbers 500, 1000, 2000, 4000 and 8000. These images are directly visualized and saved from *DPDAK*. For the following analysis we concentrate on the evolution of the intensity at the critical angles of the materials involved (Müller-Buschbaum, 2003 ▶) and that of the side maxima, corresponding in real space to the most prominent in-plane distance of the Au clusters on the surface. Purely qualitatively, the side maxima are shifted from higher to lower values in reciprocal space, meaning an increase of this length scale during the deposition process is observed.

**Figure 6:**
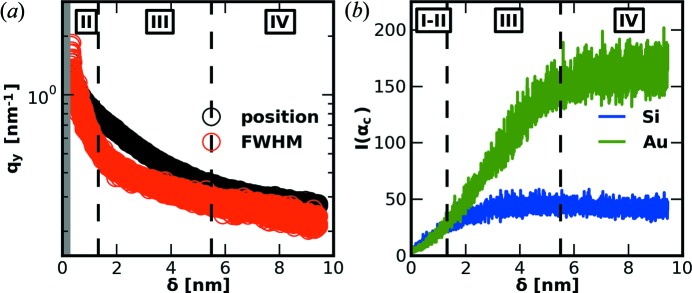
(*a*) Peak position (black) and FWHM (red) for frames 250–3150 from Fig. 5 ▶(*c*). The dashed lines indicate several kinetic transition points between different growth stages (I–IV). (*b*) Intensity at the corresponding exit angles α_c_ of Au (green) and Si (blue).

In more detail, we visualize as a color map in Fig. 5 ▶(*b*) the horizontal line cuts of the two-dimensional GISAXS data performed using *DPDAK* at the position indicated by the dashed line in Fig. 5 ▶(*a*) as function of frame number. This color map as saved from *DPDAK* shows the continuous growth of the Au clusters. The additional modulations stem from the growing height of the clusters. In order to extract this length scale quantitatively, we fitted two Lorentzian functions to every line cut using *DPDAK*. A single cut and the corresponding fit result from *DPDAK* is shown as an inset in Fig. 5 ▶(*b*) and the resulting map of fits in Fig. 5 ▶(*c*). Please note that the two-dimensional images and the color maps were saved from *DPDAK*, while the inset and the labels as well as the dashed lines were subsequently incorporated using standard graphics software. The individual parameter position and FWHM (full width at half-maximum) of the Lorentzian fits of the side maxima are shown in Fig. 6 ▶(*a*). Their evolution is related to the calculated deposited thickness of the Au layer. In addition, we present the intensity evolution of the Yoneda regions (Yoneda, 1963 ▶; Müller-Buschbaum, 2003 ▶) at the critical angles α_c_(Au) and α_c_(Si) in Fig. 6 ▶(*b*). Note that Figs. 6 ▶(*a*) and 6 ▶(*b*) use the parameters extracted from *DPDAK* and were plotted using standard imaging software to visualize the change of kinetics. The detailed behavior indicates a transition in the porosity and coverage of the silicon wafer. More details are given by Schwartzkopf *et al.* (2013 ▶).

**Figure 7:**
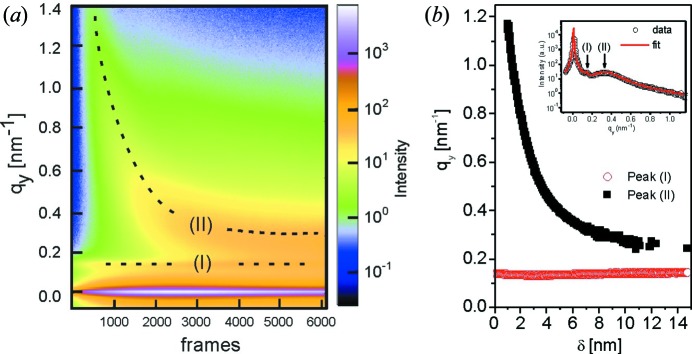
(*a*) Mapping of the horizontal line cuts (*q_y_*) collected from 0.1 s time-resolved two-dimensional GISAXS data. Intensity peaks related to the polymer template (I) and metal clusters (II) are indicated. (*b*) *q_y_* values of the position of peak (I) and peak (II) as a function of effective metal load layer thickness δ, extracted *via* fitting of individual line cuts. The inset shows an individual fit of frame 3000 as obtained by the *DPDAK* software.

During another experiment at beamline MiNaXS/P03 of DESY, gold in its atomic phase was deposited on a polymer template. Such metal deposition on polymer templates is a powerful way for achieving nanostructured hybrid films (Metwalli *et al.*, 2008 ▶, 2013 ▶). The template was a diblock copolymer film with a cylindrical microphase separation structure [polystyrene-block-polybutadiene, P(S-*b*-B), *M*_w_ = 104.7 kg mol^−1^ and a volume fraction *f*_ps_ = 0.32, resulting in a special surface morphology. A sputtering rate of 1.44 ± 0.09 nm min^−1^ was chosen, and two-dimensional GISAXS data were acquired *in situ* during the gold sputtering. Six thousand frames were collected by a PILATUS3 1M detector in 600 s (10 frames per second) using a microfocused X-ray beam with a beam size of 31 × 19 µm and a wavelength of λ = 96.0 ± 0.2 pm, an incidence angle of α_i_ = 0.452° and a sample-to-detector distance of *D*_SD_ = 3518 mm. To get quantitative information on the growing metal particle size and distribution, horizontal line cuts of the two-dimensional GISAXS data were performed at an average value of the Yoneda peaks of the materials involved. These horizontal line cuts (*q_y_*) were collected and simultaneously fitted using the *DPDAK* software for all collected frames. Lorentzian peak fits were employed for all scattering peaks. In this approach, *DPDAK* enabled the identification of two characteristic peaks immediately: the polymer peak (I) and the metal peak (II). Prior to the gold deposition, a single characteristic peak (0.141 nm^−1^) caused by the polymer template was observed. As seen from Fig. 7 ▶, the position of the scattering peak related to the polymer (I) stays at a fixed *q_y_* position (0.141 nm^−1^) upon gold deposition. In contrast, the metal characteristic peak (II) shifts from a very large *q_y_* value to lower *q_y_* values during metal deposition (Fig. 7 ▶). This evolution in reciprocal space means an increase of the corresponding real-space distance between the metal particles with increasing sputter time. It reveals a continuous particle coalescence forming large particles. At a metal load of about 4.4 nm, the latter peaks (II) level off at a *q_y_* value of 0.281 nm^−1^. At this metal load (4.4 nm), a quasi-uniform metal layer on top of the polymer film is assumed. With the help of the *DPDAK* software, plotting and simultaneous fitting of all scattering peaks in the 6000 frames can be performed efficiently in a single processing step.

The last example shows how to map orientation parameters from scanning SAXS, characterizing microfluidic experiments. Such *in situ* experiments can help to understand flow phenomena in nature and technology. Related examples from nature would be the flow of proteins or blood cells in confined geometries (Pfohl *et al.*, 2007 ▶) or the formation of natural fibers, such as spider dragline silk fibers, where well formed nanostructures lead to strong mechanical properties (Glišović *et al.*, 2008 ▶). A technology-related example where material properties depend on the orientation and alignment of nanostructured domains can be found in industrial processes, such as fiber spinning involving polymers and anisotropic particles (Vad *et al.*, 2013 ▶; Trebbin *et al.*, 2013 ▶; Taheri *et al.*, 2012 ▶; Håkansson *et al.*, 2014 ▶). The understanding of particle orientation in confined geometries is of particular interest because the control of orientation on the nanoscale can be leveraged to improve macroscopic material properties without the need to replace the material itself (Vad *et al.*, 2013 ▶; Trebbin *et al.*, 2013 ▶; Håkansson *et al.*, 2014 ▶).

In order to study these fundamentals, the flow of polymeric wormlike micelles in tapered microchannels of X-ray-compatible microfluidic devices has been investigated at beamline MiNaXS/P03 at PETRA III. These anisotropic micelles serve as a model system for anisotropic proteins, fibrous structures or elongated particles, while the microchannels represent confined geometries, such as spinning dyes or blood vessels. This double model approach allows us to precisely control relevant experimental parameters, such as the sample rheology, the geometry, or the shear and extensional force field, and to study their effect on the orientation of anisotropic samples. The observed time component is a function of the measurement location. Hence, time-resolved *in situ* studies, such as dynamic processes or nucleation and growth kinetics, can be measured with very high temporal resolution and down to microsecond timescales (Knight *et al.*, 1998 ▶; Pollack *et al.*, 2001 ▶; Russell *et al.*, 2002 ▶). In this example, an aqueous solution of the wormlike micelles was pumped at a flow rate of 100 µl h^−1^ through a tapered microchannel. The microfluidic channel had a width of 333 µm and a 44.4 µm tapering (7.5:1 ratio) at a constant channel height of 100 µm (in the beam direction). The microchannel was mapped with a microfocused X-ray beam spot size of 20 × 20 µm at an energy of 13 keV with a scan grid of 14 × 54 positions (630 positions). Further technical details can be found in the original article (Trebbin *et al.*, 2013 ▶).

**Figure 8:**
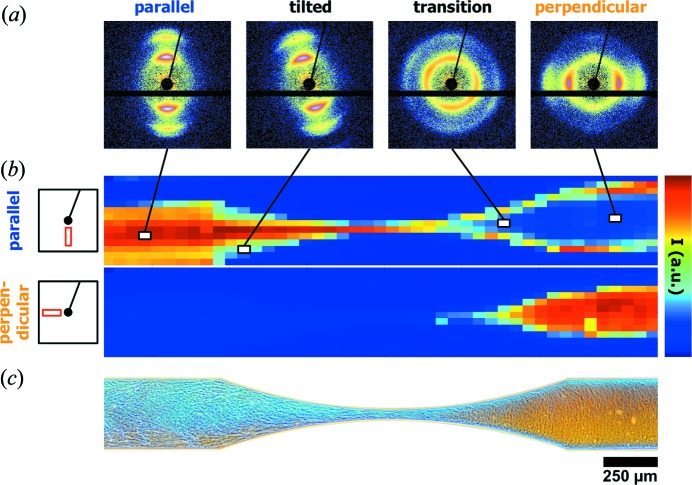
The flow of wormlike polymeric micelles can be studied using SAXS by scanning the microfluidic channel with a microfocused X-ray beam. (*a*) The measured SAXS patterns reveal micelles in parallel, tilted, transitional and perpendicular orientations depending on their location, as indicated by the straight lines. (*b*) The information from these SAXS patterns is used to generate color maps in real time using *DPDAK*. Each of these pixels is color coded on the basis of the averaged ROI of the parallel or the perpendicular micelle orientation. (*c*) The SAXS results are also in good agreement with the corresponding polarization microscopic images that are based on the samples birefringence. Here, the blue color represents a parallel micelle orientation with respect to the flow, while the orange color indicates a perpendicular orientation.

This experimental setup allows us to demonstrate the benefit of using tailored *DPDAK* plug-ins for online analysis. The large number of scattering patterns obtained from scanning the microfluidic channels were processed using *DPDAK* by first correcting the SAXS patterns for background signals. Next, for radial and azimuthal averaging of the two-dimensional scattering images, an ROI was chosen that corresponded to either parallel or perpendicular micelle orientation, as indicated in Fig. 8 ▶(*b*, left). These ROIs were then averaged and plotted as color-coded pixels for each scattering pattern of the scan grid. The resulting pixel maps, as shown in Fig. 8 ▶(*b*), are generated in real time during the microfluidic SAXS experiments, revealing orientation information on the flow of the micelles. It is also important to note that each pixel represents a full two-dimensional scattering pattern containing relevant structural information about the sample, such as for example orientation distribution, micelle diameter, unit-cell size *etc*. *DPDAK* is capable of processing the data at high speed, which allows the experimentalist to control experimental conditions and to monitor the measured and analyzed results in real time during the data acquisition. The here presented SAXS experiments reveal that the initially parallel oriented wormlike micelles were oriented perpendicularly to the flow after passing the narrow section. By coupling the results from the SAXS measurements with other methods (PolMik, μPIV and CFD simulation), it could be shown that the reorientation of the anisotropic sample depended on the ratio between shear and extensional flow (Trebbin *et al.*, 2013 ▶).

## 6. Conclusions and outlook   

*DPDAK* was developed to perform simple but fast online data reduction and analysis, allowing the user to review scattering data for quality control and to follow a number of parameters of interest predefined by the user as a function of the scanning parameters. The procedures typically imply basic corrections (*e.g.* beam intensity corrections, background subtraction, calibration *etc*.) and the extraction of scalar parameters from the two-dimensional patterns, such as peak positions or widths, moments of the intensity distribution and their ratio, power law exponents, and integrated intensities. These parameters can then be visualized by *DPDAK* in an efficient way (typically as an image or a profile as a function of the scanning parameters), allowing an interactive experiment control by adjusting the experimental protocol accordingly. We therefore believe a huge amount of precious synchrotron beamtime can be saved by using *DPDAK*.

The *DPDAK* platform is independent of the beamline and/or experiment control software so that it can be exchanged between different groups working with synchrotron data. The current version of the software package *DPDAK* and an up-to-date manual reside on a dedicated web site (https://dpdak.desy.de/). The concept of *DPDAK* allows the treatment of many kinds of data, *i.e.* *DPDAK* can handle data from different types of experiments, like the presented examples based on WAXS, SAXS, X-ray fluorescence and GISAXS, as well as general curve fitting. Owing to the easy to use plug-in interface it is possible to include further data reduction and analysis methods. For example, the *pySAXS* package (Taché *et al.*, 2013 ▶) could be included to support specialized model fitting within *DPDAK*. Therefore plug-ins for each model in the *pySXAS* package would need to be developed. In future, the use of parallel computing in terms of graphics and multicore processors can also be envisaged, the *HiPGISAXS* software being a very recent example of this (Chourou *et al.*, 2013 ▶).

## Figures and Tables

**Table 1:** Comparison of a selection of available data analysis tools

	Open source	Online mode	Macro/plug-in	Operating system	Reference
*BioXTAS RAW*	GPL	Yes	No	Win/Linux	Nielsen *et al.* (2009 ▶)
*XRDUA*	GPL	Yes	No	Win/Linux	http://xrdua.ua.ac.be/
*SAXSutilitie*s	GPL	Yes	No	Win/Linux	http://www.sztucki.de/SAXSutilities/
*pySAXS*	CeCILL	No	API†	Win/Linux	Taché *et al.* (2013 ▶)
*Sasfit*	GPL	No	Yes	Win/Linux	Kohlbrecher & Bressler (2011 ▶)
*Scatter* ‡	No	No	No	Win	Förster *et al.* (2010 ▶)
*DAWN*	Yes	No	Yes	Win/Linux/Mac	http://www.dawnsci.org/

† *pySAXS* is designed as a Python application programming interface (API) and can be used in any data analysis software. New analysis methods can be added to the API. It also implements a user interface which is currently not designed for online processing.

‡ *Scatter* is primarily designed for the analysis, modeling and fitting of one- and two-dimensional small-angle scattering data of non-ordered, partially ordered or fully ordered nano- and mesoscale structures (Förster *et al.*, 2010 ▶).
